# Hemangioblastoma of the pons and middle cerebellar peduncle

**DOI:** 10.3171/2019.10.FocusVid.19402

**Published:** 2019-10-01

**Authors:** Satoshi Kiyofuji, Harry J. Cloft, Colin L. W. Driscoll, Michael J. Link

**Affiliations:** 1 Departments of Neurologic Surgery,; 2 Radiology, and; 3 Otorhinolaryngology, Mayo Clinic, Rochester, Minnesota

**Keywords:** cerebellopontine angle, hemangioblastoma, retrosigmoid approach, tumor embolization, video

## Abstract

A 60-year-old man with a history of four prior operations for a left cerebellar/middle cerebellar peduncle hemangioblastoma presented with hearing loss, imbalance, and ataxia (de la Monte and Horowitz, 1989). Magnetic resonance imaging (MRI) demonstrated a 3-cm cystic mass with heterogeneous enhancement in the same location. We resected the mass via reopening of the retrosigmoid approach (Lee et al., 2014). Left cranial nerves IV, V, VII, VIII, IX, X, and XI were all well identified and preserved, and feeding arteries from the brainstem were meticulously coagulated and transected without violating the tumor-brainstem interface (Chen et al., 2013). Preoperative embolization greatly aided safe resection of the mass, whose pathology revealed recurrence of hemangioblastoma (Eskridge et al., 1996; Kim et al., 2006; Sakamoto et al., 2012).

The video can be found here: https://youtu.be/3mZgY15xOZc.

## Transcript

This video demonstrates microsurgical resection of hemangioblastoma in the pons to middle cerebellar peduncle. A 60-year-old man with a history of four prior surgical resections of a left cerebellar/middle cerebellar peduncle hemangioblastoma underwent follow-up MRI in 2014, 20 years after the most recent surgery, which revealed no evidence of residual/recurrent tumor. Five years later, he presented for the first time to our institution with imbalance, hearing loss, and vertigo. MRI demonstrated a 3-cm cystic mass in the pons and left middle cerebellar peduncle. Neurologically, he had decreased hearing on the left side and mild ataxia. Angiogram demonstrated feeding from the left superior cerebellar artery, and anterior inferior cerebellar artery, suggesting recurrence of hemangioblastoma. Branches of the left SCA and AICA were embolized the day before surgery. In surgery, the patient was positioned in the right lateral decubitus position, and left retrosigmoid approach via reopening of the previous left suboccipital craniectomy was performed. We monitored cranial nerves V, VII, VIII, X, and XI on the left side. The previous retroauricular curvilinear incision was reopened to expose the dura. The inferior part of the dura was opened, and CSF was drained out to release pressure. The dura was further opened in a flap fashion and retracted laterally. The lateral medullary cistern was opened, and the lower cranial nerves were identified. A scarred tissue was resected to further open the lateral pontine cistern. The lower cranial nerves were released from the cerebellum. Cranial nerves XI and X were stimulated and identified. The facial nerve was also stimulated and identified. We released the arachnoid above the VII and VIII complex. The VII and VIII complex was densely adherent to the tumor, and this was released with multiple Rhoton dissectors. The bleeding from VII and VIII complex was controlled with Gelfoam. The tumor was lifted off from the cerebellum. The feeding arteries from the cerebellum and brainstem were meticulously coagulated and transected. Here, we identified the branch of AICA that was embolized the day before. This was coagulated and transected from the tumor. This is the superior part of the tumor. As we lifted the tumor off from the cerebellum, attention was paid not to violate the tumor interface. This is the branch of SCA that was embolized. As we dissected the arachnoid near the tentorium, the fifth nerve was identified underneath. The superior petrosal vein was coagulated and transected. The part of the tumor extending into the internal auditory canal was dissected from the VII and VIII complex. This was coagulated and released from the main component of the tumor. As we elevated the tumor inferiorly to superiorly, another branch of AICA that was embolized was identified. This was coagulated and transected. Then the tumor was dissected off from the fifth nerve. Finally, the tumor was dissected off from the middle cerebellar peduncle. Here, from superiorly to inferiorly, we identified cranial nerves IV under the tentorium, motor root of V, sensory roots of V, VII, and VIII complex, and lower cranial nerves. The posterior wall of the internal auditory canal was drilled with ultrasonic aspirator. The dura of the internal auditory canal was opened sharply. The residual tumor in the internal auditory canal, and the superior vestibular nerve was dissected off from the facial nerve. The facial nerve was identified with electrical stimulation. The small residual tumor in the internal auditory canal was transected sharply with the superior vestibular nerve. The wall of the internal auditory canal was packed with bone wax and Surgicel. The facial nerve was stimulated with a good response at the end of procedure. The dura was closed watertight with 3-0 Nurolon and 6-0 Prolene stitches. The wound was closed in multiple-layer fashion. Postoperatively, he had slightly decreased sensation in the left V3 distribution, and no hearing on the left side. He denied any facial weakness or dysphagia. He was discharged home on postoperative day 2 with baseline left hemibody ataxia.

## Time points

1:36 Opening of the dura
2:09 Identification of the lower cranial nerves
2:35 Dissection of VII and VIII complex
4:10 Identification of fifth nerve
5:37 Completion of resection of cisternal component of the tumor
6:08 Drilling of the posterior wall of the internal auditory canal (IAC)
6:26 Opening of the dura of IAC
7:09 Resection of tumor in IAC
7:49 Closure of the dura

## References

[1] Chen LF Yang Y Yu XG Bu B Xu BN Zhou DB Operative management of brainstem hemangioblastomas J Clin Neurosci 20 1727 1733 2013 24055208 10.1016/j.jocn.2013.01.027

[2] de la Monte SM Horowitz SA Hemangioblastomas: clinical and histopathological factors correlated with recurrence Neurosurgery 25 695 698 1989 2586723

[3] Eskridge JM McAuliffe W Harris B Kim DK Scott J Winn HR Preoperative endovascular embolization of craniospinal hemangioblastomas Am J Neuroradiol 17 525 531 1996 8881249 PMC8337986

[4] Kim LJ Albuquerque FC Aziz-Sultan A Spetzler RF McDougall CG Low morbidity associated with the use of NBCA liquid adhesive for preoperative transarterial embolization of central nervous system tumors Neurosurgery 59 98 104 2006 10.1227/01.neu.0000243288.91167.43 28180606

[5] Lee B Marquez YD Giannotta SL Resection of a cystic brainstem hemangioblastoma via a retrosigmoid approach Neurosurg Focus 36 1 Suppl 1 2014 10.3171/2014.V1.FOCUS13434 24380524

[6] Sakamoto N Ishikawa E Nakai Y Akutsu H Yamamoto T Nakai K Preoperative endovascular embolization for hemangioblastoma in the posterior fossa Neurol Med Chir (Tokyo) 52 878 884 2012 23269042 10.2176/nmc.52.878

